# XAV939, a tankyrase 1 inhibitior, promotes cell apoptosis in neuroblastoma cell lines by inhibiting Wnt/β-catenin signaling pathway

**DOI:** 10.1186/1756-9966-32-100

**Published:** 2013-12-05

**Authors:** Xiao-Hong Tian, Wei-Jian Hou, Yan Fang, Jun Fan, Hao Tong, Shu-Ling Bai, Qu Chen, He Xu, Yan Li

**Affiliations:** 1 Department of Tissue Engineering, College of Basic Medical Sciences, China Medical University, Shenyang 110001, PR China; 2 Department of Anatomy, Liaoning Medical University, Jinzhou 121001, PR China; 3 The Seven-year-system of China Medical University, Shenyang 110001, PR China; 4 English Teaching and Research Section for Graduate, China Medical University, Shenyang 110001, Liaoning Province, PR China

**Keywords:** Neuroblastoma, Tankyrase 1, Wnt signaling, XAV939

## Abstract

**Background:**

Neuroblastoma (NB) is the most common extracranial solid tumor in childhood. The present treatment including surgery, chemotherapy and radiation, which have only 40% long-term cure rates, and usually cause tumor recurrence. Thus, looking for new effective and less toxic therapies has important significance. XAV939 is a small molecule inhibitor of tankyrase 1(TNKS1). The objective of this study is to investigate the effect of XAV939 on the proliferation and apoptosis of NB cell lines, and the related mechanism.

**Methods:**

In the present study, we used both XAV939 treatment and RNAi method to demonstrate that TNKS1 inhibition may be a potential mechanism to cure NB. MTT method was used for determining the cell viability and the appropriate concerntration for follow-up assays. The colony formation assay, Annexin V staining and cell cycle analysis were used for detecting colony forming ability, cell apoptosis and the percentage of different cell cycle. The Western blot was used for detecting the expression of key proteins of Wnt/ beta-catenin (Wnt/β-catenin) signaling pathway.

**Results:**

The results showed that TNKS1 inhibition decreased the viability of SH-SY5Y, SK-N-SH and IMR-32 cells, induced apoptosis in SH-SY5Y as well as SK-N-SH cells, and led to the accumulation of NB cells in the S and G2/M phase of the cell cycle. Moreover, we demonstrated TNKS1 inhibition may in part blocked Wnt/β-catenin signaling and reduced the expression of anti-apoptosis protein. Finally, we also demonstrated that TNKS1 inhibition decreased colony formation in vitro.

**Conclusions:**

These findings suggested that TNKS1 may be a potential molecule target for the treatment of NB.

## Background

Neuroblastoma (NB) is the most common extracranial solid tumor in childhood and infancy, which is an embryonic tumor of the postganglionic sympathetic nervous system. It remains an important pediatric problem because it accounts for 8–10% of all childhood cancers and for approximately 15% of cancer deaths in children [1-3]. It is associated with poor prognosis because of its ability to regress spontaneously, transform, or show aggressive behavior [4]. Current treatment for high-risk NB consists of a coordinated sequence of chemotherapy, surgery, and radiation [5,6]. Even with this aggressive treatment, less than 40% of children are likely to achieve long-term cure [5-7]. After that the patients usually underwent tumor recurrence as well as long-term complications following high-dose chemotherapy [8,9]. There is an urgent need for more effective and less toxic therapies, and molecular target-directed drugs are potential representation.

The evolutionarily conserved Wnt/beta-catenin (Wnt/β-catenin) pathway, which is well-described and canonical, is related to human birth defects, cancer, and other diseases [10]. Wnt signal pathway is one of the fundamental mechanisms that regulate cell proliferation, cell polarity and cell differentiation during embryonic development [11]. As a result, inappropriate regulation of Wnt signaling occurs in several types of cancer, including colon, liver and brain tumors of neuroectodermal origin [10]. Whether the Wnt/β-catenin pathway is activated or not depends on the stability of β-catenin in the cytoplasm. β-catenin is regulated by a destruction complex, which is composed of the scaffolding protein Axin, the tumor suppressor adenomatous polyposis coli gene product (APC), casein kinase 1, and glycogen synthase kinase 3(GSK3). In the absence of Wnt stimulation, β-catenin is phosphorylated by the complex and degraded by the ubiquitination/proteasome pathway. In the presence of Wnt, the Axin-mediated β-catenin phosphorylation can be inhibited, then, accumulated β-catenin enters the nucleus and binds to the TCF/LEF family of DNA-binding factors for activation of Wnt pathway-responsive gene transcription, such as cyclin D1, c-*myc*, axin2 and so on [10,12]. Inhibition of Wnt signaling has become an attractive strategy for cancer therapeutics [13].

An exciting study published recently in Nature [14], together with an earlier one [15], has verified a new class of small molecule inhibitors, XAV939, which could block Wnt signaling in colon cancer cell lines by binding to tankyrase (TNKS) catalytic poly-ADP-ribose polymerase (PARP) domain, and then resulted in dramatic stabilization of the Axin protein, thereby lead to increased β-catenin destruction. As a major member of the TNKS family, it has been reported that tankyrase 1(TNKS1) were up-regulated in a variety of cancers, including multiple myeloma, plasma cell leukemia, high-grade non-Hodgkin’s lymphomas, breast cancer, colon cancer, and bladder cancer [16-22]. These reports suggested that TNKS1 played a role in tumor progression. Recently, Bao R *et al.* demonstrated that XAV939 or siRNA-mediated abrogation of TNKS expression increases Axin1 and Axin2 protein levels and attenuates Wnt-induced transcriptional responses in several breast cancer lines [23]. In our previous studies, we have known that TNKS1 was also up-regulated in NB SH-SY5Y cells (data not shown). It has also been reported that the β-catenin has a close relationship with the prognosis of NB. The stronger the β-catenin expressed in nucleus, the higher risk of NB would be, and the worse the prognosis was [24]. However, whether the proliferation of NB cell lines could be inhibited through blocking the Wnt pathway or other mechanisms?

In the present study, we have investigated the anti-proliferative effect of XAV939 on the human NB cell lines. In addition, we studied the cell apoptosis induced by XAV939 and assessed the role of Wnt signaling in it.

## Materials and methods

### Cell culture and TNKS1 inhibitor

Human NB SH-SY5Y, SK-N-SH and IMR-32 cells were obtained from the American Type Culture Collection (ATCC; Rockville, USA). Cells were maintained in Dulbecco’s modified Eagle’s medium (DMEM; Hyclone), with 10% fetal bovine serum (FBS; Gibco) and 1% penicillin/streptomycin (Sigma Chemical Co., St Louis, Missouri) and were grown in a 5% CO_2_ incubator at 37°C. The TNKS1 inhibitor XAV939 was purchased from Sigma Aldrich.

### Assessment of cellular viability

Cellular viability was assessed by MTT method. Briefly, equal numbers of NB SH-SY5Y, SK-N-SH and IMR-32 cells were plated at a density of 1 × 10^4^ per well in 96-well plates, and were treated with various concentrations of XAV939 for 24, 48, or 72 h. 20 μl MTT (5 mg/ml) were incubated with cells of each sample for 4 h, then were replaced with 150 μl DMSO and 96-well plates were rotated gently for 10 min. Cell viability was determined by measuring colorimetric absorbance at 490 nm, and was read with a microplate reader [25]. Experiments were done in triplicate and average activity rates relative to control and standard errors were calculated.

### Colony formation assay

Colony formation assays were performed as described [26]. Briefly, SH-SY5Y cells were plated in triplicate at 100 cells per well in 6-well plates and cultured in DMEM medium supplemented with 10% FBS. After 4-5 h, cells were treated with DMSO or XAV939, as well as transfected with lentivirus-mediated scrambled-shRNA (SCR group) or TNKS1-shRNA (shRNA group). Colonies were allowed to form for 14 days and fixed in methanol for 15 minutes, and dyed with crystal violet for 15 minutes at room temperature. Afterward, the dye was washed off and colonies that contained more than 50 cells were counted. The colony formation efficiency was the ratio of the colony number to the planted cell number.

### Apoptosis assays

Apoptosis was measured using Annexin V/FITC Apoptosis Detection kit (KeyGEN Biotech, Nanjing, China) following the manufacturer’s protocol. Briefly, equal numbers of SH-SY5Y and SK-N-SH cells, treated with DMSO or XAV939 for 24, 48, or 72 h, were incubated with Annexin V-FITC, followed by staining of their DNA with propidium iodide (PI) in the dark. Then, each sample was analyzed by fluorescence-activated cell sorting (FACS) (BD, San Jose, CA, USA). The percentages of cells staining positive for Annexin V were calculated, and means as well as standard error were plotted. Alternatively, apoptosis was also determined using Hoechst 33342 staining. After treatment, cells were washed with PBS and stained with Hoechst 33342 (10 μg/mL, Sigma Aldrich). Then the cells were observed by fluorescent microscope (Olympus Inverted Fluorescence Microscope, I × 71) with excitation at 340 nm and approximately 100 cells from five random microscopic fields were counted. The percentage of apoptotic cells was calculated as the ratio of apoptotic cells to total cells. Mean and standard error were calculated for each time point and treatment group.

### Cell cycle analysis

Equal numbers of SH-SY5Y, SK-N-SH and IMR-32 cells were plated in 10 cm dishes and treated with DMSO or XAV939 for 24, 48, or 72 h. 10^6^ cells were trypsinized, fixed with 70% ethanol, and incubated over night at 4°C, then were incubated in 100 μl RNase at 37°C for 30 min, followed by staining of their DNA with 400 μl PI for 30 min in the dark, and analyzed by FACS. The average percentages of cells in G0/G1, S or G2/M phases of the cell cycle were quantified and standard error was calculated for three experiments.

### Western blot

Equal numbers of SH-SY5Y and SK-N-SH cells were plated on 10 cm dishes and treated with DMSO or XAV939 for 24, 48 or 72 h. Then the cells were lysed with RIPA buffer and protein concentration was determined by the Bradford method. Equal amounts of protein (40 μg) were used for Western blot analysis with antibodies to anti-β-catenin (Santa Cruz, sc-7199), anti-Cyclin D1 (Santa Cruz, sc-718), anti-c-Myc (Santa Cruz: sc-789) and anti-Bcl-2 (Santa Cruz, sc-492). Specific antibody binding was detected by horseradish peroxidase-conjugated goat anti-rabbit antibodies and visualized with ECL reagent (Santa cruz) according to the manufacturer’s protocol. Antibody to actin was used to evaluate protein loading in each lane.

### Silencing of TNKS1 with shRNA

To identify shRNA sequences could knockdown TNKS1 in SH-SY5Y and SK-N-SH cells, we screened three MISSION shRNA clones NM_003747 (GENECHEM CO., LTD., Shanghai, China) targeted against the human TNKS1 sequence. MISSION shRNA clones together with packaging and envelope plasmids GV118 (GENECHEM CO., LTD., Shanghai, China), were transfected into HEK 293 T packaging cells using Lipofectamine 2000 (Invitrogen). At 48 h post-transfection, virus-containing media was used to infect NB cell lines. GFP was used to monitor the efficiency of HEK 293 T transfection and infection. After selection with puromycin (5 μg/ml) for 48 h, cells were tested for TNKS1 expression by qRT-PCR and then used for clonogenic survival assays and Western blot analyses.

### Statistical analysis

The results were presented as Mean ± Standard deviation (S.D.) of triplicate determinations. The data were processed using the Statistical Package for the Social Sciences, version 16.0 (SPSS Inc., Chicago, IL, USA). One-way ANOVA was performed for comparison between different groups. Dunnett’s t (when homogeneity of variances existed) or Dunnett T3 (when heterogeneity of variances existed) was calculated. A P-value of < 0.05 was regarded as statistically significant difference.

## Results

### TNKS1 inhibition decreases cell growth and proliferation in NB cell lines

**Figure 1:**
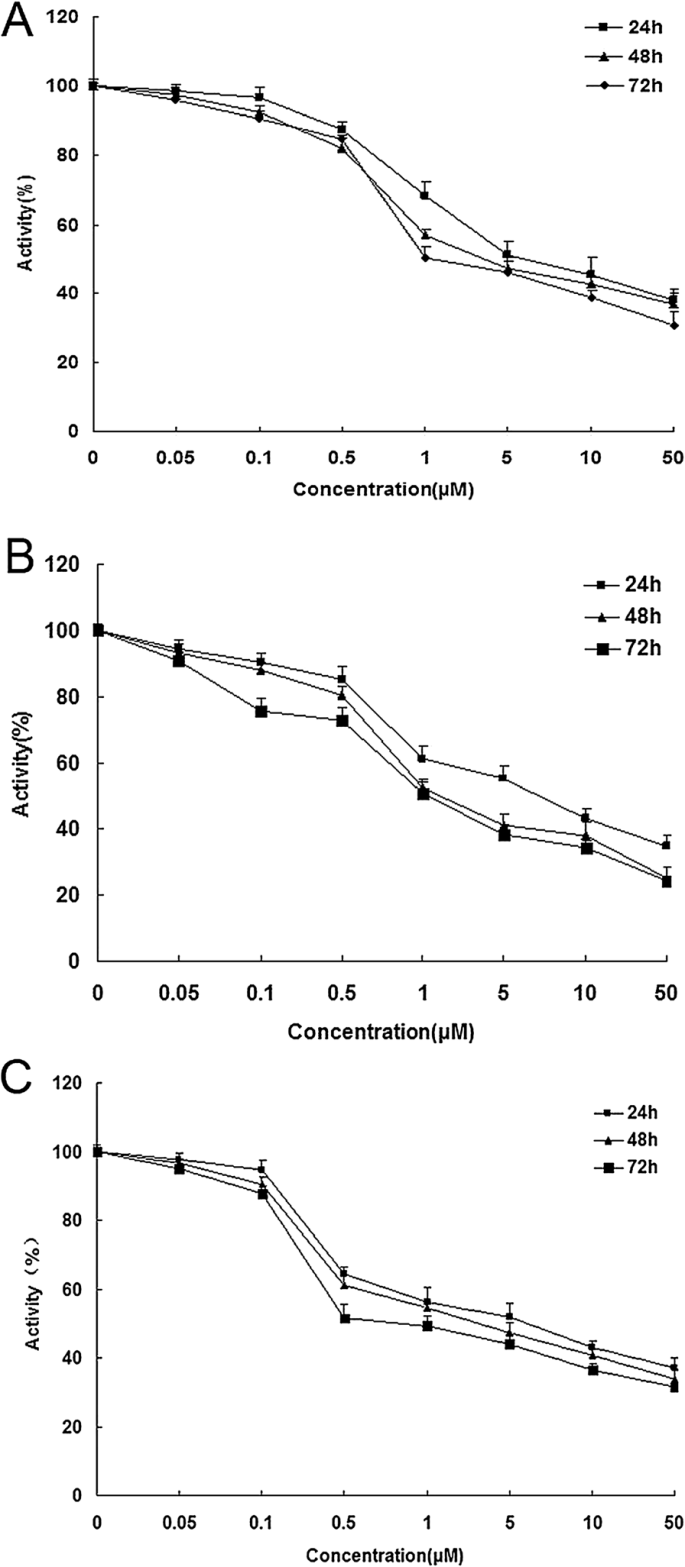
**The cellular activity of SH-SY5Y, SK-N-SH and IMR-32 cells after XAV939 treatment at 24 h, 48 h and 72 h. A.** The cellular activity of SH-SY5Y cells. **B.** The cellular activity of IMR-32 cells. **C.** The cellular activity of SK-N-SH cells. P < 0.05.

XAV939 has been described as a potent, small molecule inhibitor of TNKS1 and 2 and could inhibit the growth of DLD-1 cancer cells [14]. To elucidate the role of XAV939 in NB, we investigated how XAV939 affects cell proliferation in NB cell lines with different concentrations. After that, both SH-SY5Y cells and IMR-32 cells showed reduction in cell proliferation after 24 h of treatment with 1 μM XAV939, with a maximum reduction at 72 h (Figure 1A, B). However, SK-N-SH cells showed the same effect only with 0.5 μM XAV939 treatment (Figure 1C). This anti-proliferative effect was dose and time dependent at 1, 5, 10 and 50 μM at 24, 48 and 72 h. These results indicate that inhibition of TNKS1 by small molecule inhibitor attenuates NB cell proliferation. Thus 1 or 0.5 μM XAV939 were used depending on the cell lines for further assays.

### TNKS1 inhibition reduces SH-SY5Y cell survival

**Figure 2:**
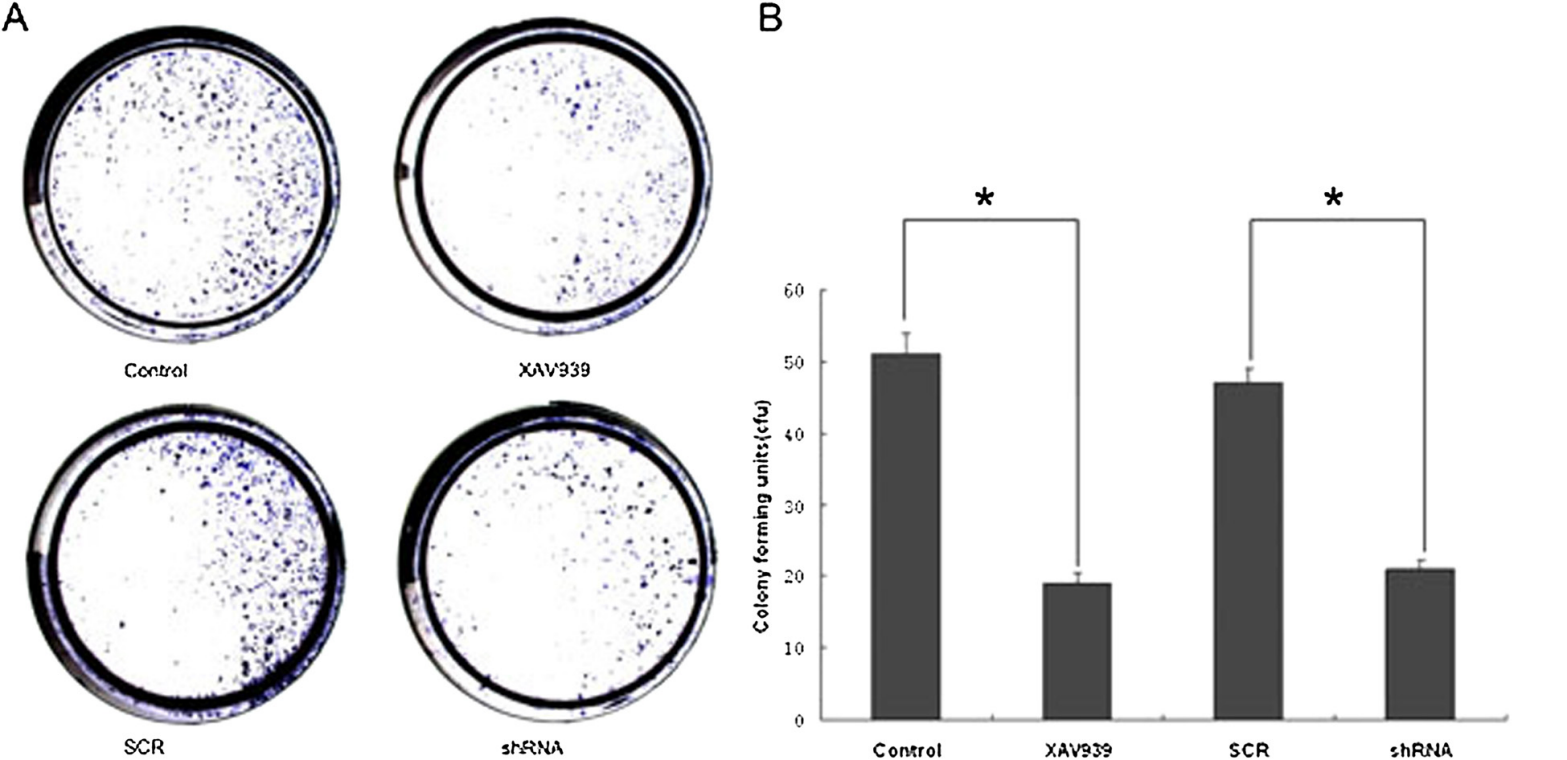
**TNKS1 inhibition induces cell death in SH-SY5Y cells. A.** The cell colony stained by 1% crystal violet in control gorup, XAV939 group, SCR group and shRNA group. **B.** The bar graph depicts the colony forming units(cfu) in different groups. *P < 0.01 compared to controls.

To determine whether TNKS1 inhibition reduces cell viability and survival of SH-SY5Y cells, we performed a colony formation assay in vitro. The number of colonies in the control and various treatment groups were counted and are summarized in Figure 2. From these results it is evident that the XAV939 caused 62.7% inhibition of colony formation in SH-SY5Y cells. In addition, we also observed the effect of shRNA for TNKS1 on cell colony formation. As shown in Figure 2, specific knockdown of TNKS1 by shRNA in SH-SY5Y cells resulted in a significant decrease (55.3%) in the number of colonies, as compared to SCR group (P < 0.01, Figure 2B). These results indicate that the growth inhibitory effects of XAV939 on SH-SY5Y cells are due to TNKS1-dependent inhibition.

### TNKS1 inhibition induces apoptosis in NB cell lines

**Figure 3:**
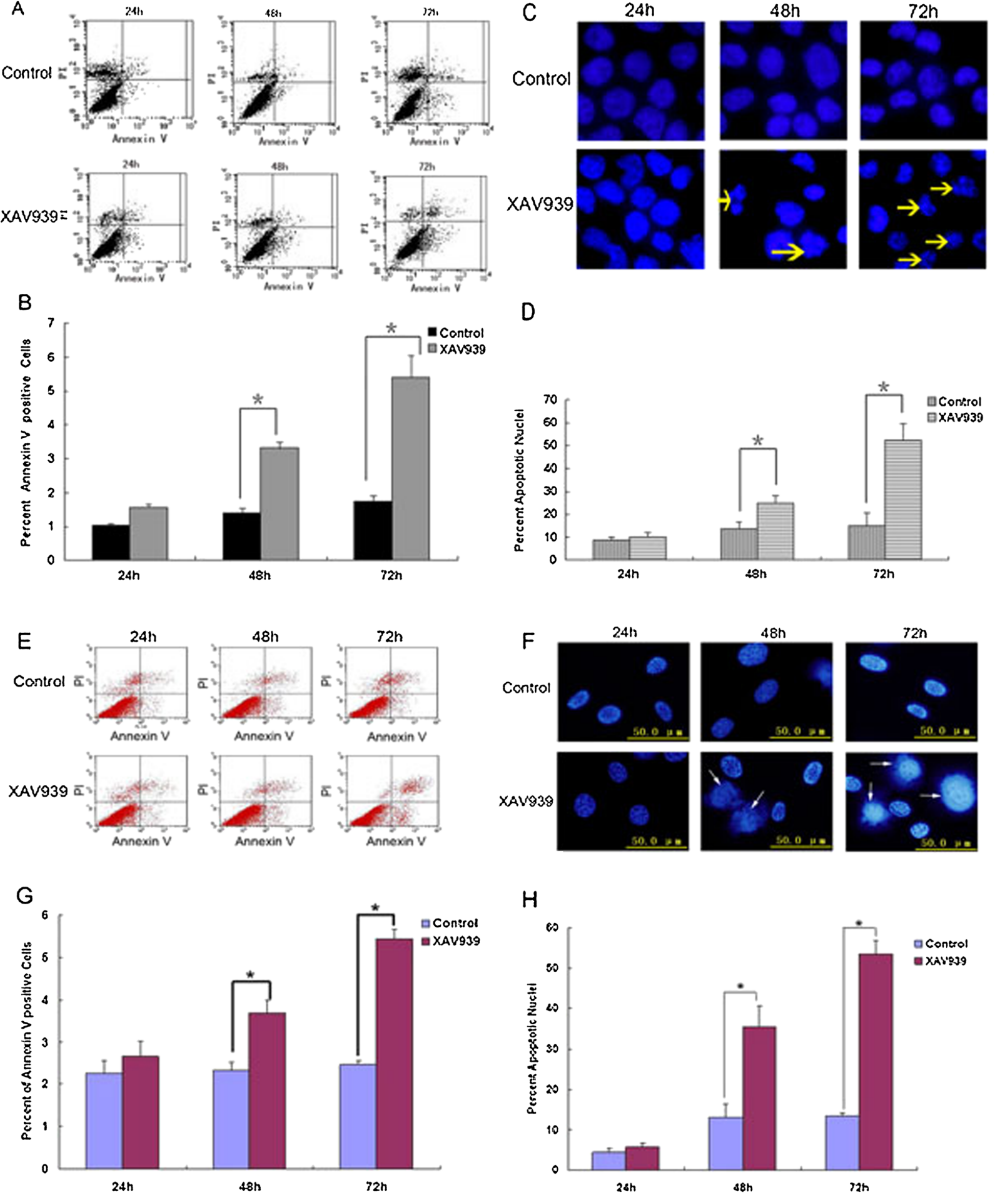
**TNKS1 inhibition induces cell apoptosis in SH-SY5Y and SK-N-SH cells. A, E.** The figures of SH-SY5Y and SK-N-SH cells stained with Annexin V in control group and XAV939 group. **B, G.** The bar graph of average percent of apoptotic cells in control group and XAV939 group. *P < 0.05 compared to controls. **C, F.** The morphology of apoptotic nuclei was observed by Hoechst 33342 staining. The arrows point at the apoptotic nuclei. **D, H.** The bar graph of percentages of apoptotic cells in control group and XAV939 group. *P < 0.05 compared to controls. The apoptosis of SH-SY5Y cells was indicated by figures **A, B, C** and **D,** while that of SK-N-SH cells was indicated by figures **E, F, G** and **H**.

Apoptosis plays an important role in both the cause and treatment of tumor [27]. The early apoptotic cells could be stainned by Annexin V, which located in the right lower quadrant (Figure 3A, E). Both the SH-SY5Y and SK-N-SH cell lines showed that no significant difference in the percentage of apoptotic cells between the control and XAV939 group for 24 h (Figure 3B, G). However, after 48 h or 72 h treatment, the apoptotic rates in XAV939 group were 3.31 ± 0.17% and 5.41 ± 0.63% respectively in SH-SY5Y cells (Figure 3B), which were significantly higher than those in control group (P < 0.05, Figure 3B). Similarly, the apoptotic rates in XAV939 group were 3.69 ± 0.31% and 5.44 ± 0.24% respectively in SK-N-SH cells (Figure 3G, P < 0.05). To further confirm that TNKS1 inhibition induced apoptosis in NB cell lines, we studied the nuclear morphology of SH-SY5Y and SK-N-SH cells following Hoechst 33342 staining (Figure 3C, F). As depicted in Figure 3C and F, control cells without XAV939 treatment were uniformly stained with and displayed equally disseminated chromatin, normal and intact cell membrane. In contrast, cells treated with XAV939 for 24, 48, or 72 h illustrated varying degrees of archetypal characteristics of apoptotic cells, including the condensation of chromatin, shrinkage of nuclei, and presence of apoptotic bodies with intense blue fluorescence (Figure 3C, F). The major findings were showed by arrows in Figure 3C, F. In SH-SY5Y cells, the percentages of cells with apoptotic nuclei in XAV939 group were 9.2%, 25.0% and 52.3% respectively at 24 h, 48 h and 72 h, which in control group were 8.8%, 13.8% and 15.0% respectively (Figure 3D). In SK-N-SH cells, The percentages of cells with apoptotic nuclei in XAV939 group were 5.7%, 35.5% and 53.5% respectively at 24 h, 48 h and 72 h, which in control group were 4.5%, 13.2% and 13.5% respectively (Figure 3H). The statistical analysis showed that there was no significant difference of apoptotic cells between the control and XAV939 groups at 24 h, but the percentages of apoptotic cells in XAV939 group were significant higher than those in control group at 48 h and 72 h respectively (P < 0.05, Figure 3D, H), confirming the induction of apoptosis following treatment. Together, these results suggest that apoptosis is promoted by TNKS1 inhibition in NB cell lines.

### TNKS1 inhibition induces the accumulation of NB cell lines at G2/M

**Figure 4:**
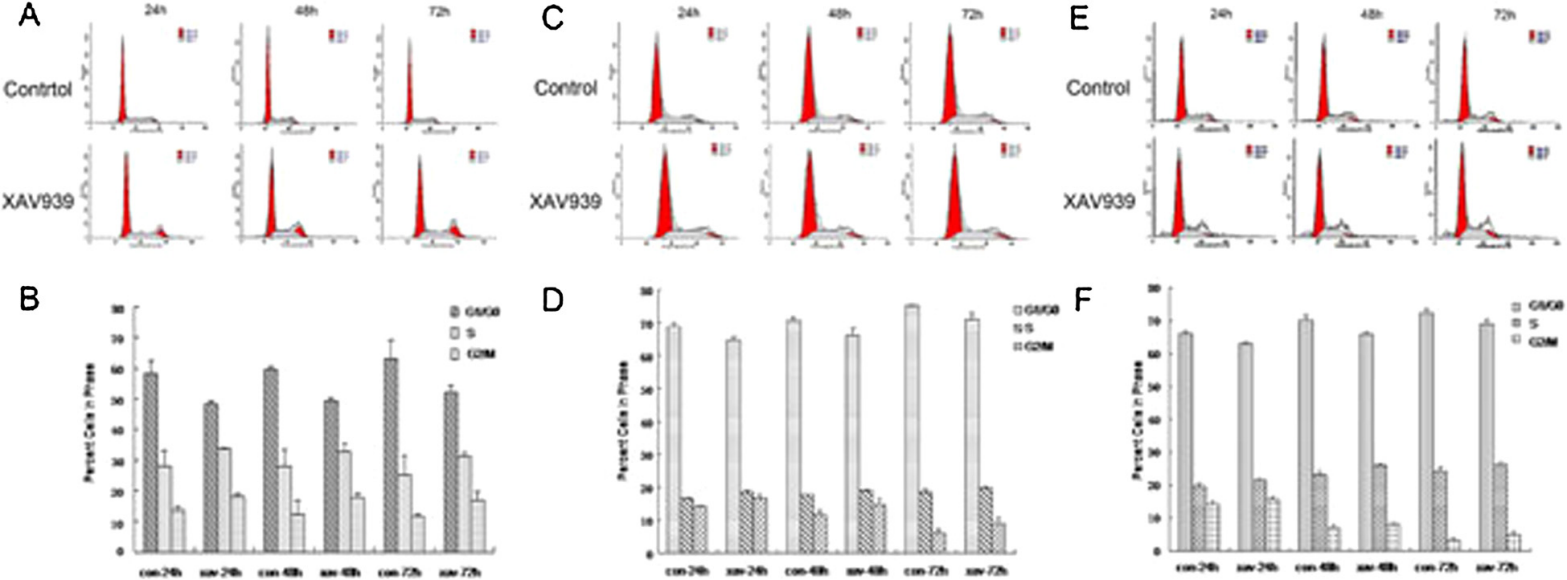
**TNKS1 inhibition induces G2/M accumulation in SH-SY5Y, SK-N-SH and IMR-32 cells. A, C, E.** The representative diagrams of distribution of stained SH-SY5Y, SK-N-SH and IMR-32 cells in control group and XAV939 group. **B, D, F.** The bar graph of the average percent cells of G0/G1, S and G2/M phases in control group and XAV939 group for SH-SY5Y, SK-N-SH and IMR-32 cells respectively (P < 0.05).

TNKS1 was proposed to be required for the resolution of sister telomere association or assembly of bipolar spindles, and TNKS1 knockdown was reported to cause strong mitotic arrest [28,29]. We detected the effect of XAV939 on cell cycle in SH-SY5Y, SK-N-SH and IMR-32 cells. As shown in Figure 4A, the number of SH-SY5Y cells in the G0/G1 phase decreased, while those in the S and G2/M phase both increased after XAV939 treatment (Figure 4A). At 24 h after treatment, 58.25% of the DMSO-treated SH-SY5Y cells were at G0/G1, 28.02% at S and 13.73% at G2/M (Figure 4B, P < 0.05). In comparison, 48.38% of the XAV939-treated SH-SY5Y cells were at G0/G1, 33.68% at S and 17.94% at G2/M (Figure 4B, P < 0.05). This trend was continued at later time points (Figure 4B). Figure 4C, E indicated the cell cycle of SK-N-SH and IMR-32 cells respectively, and showed the same tendency with that of SH-SY5Y cells (Figure 4D, F, P < 0.05). This suggested that TNKS1 plays a role in cell cycle regulation and that TNKS1 inhibition induces an accumulation of NB cell lines at G2/M and S phase of the cell cycle.

### TNKS1 inhibition reduces the expression of anti-apoptotic proteins

**Figure 5:**
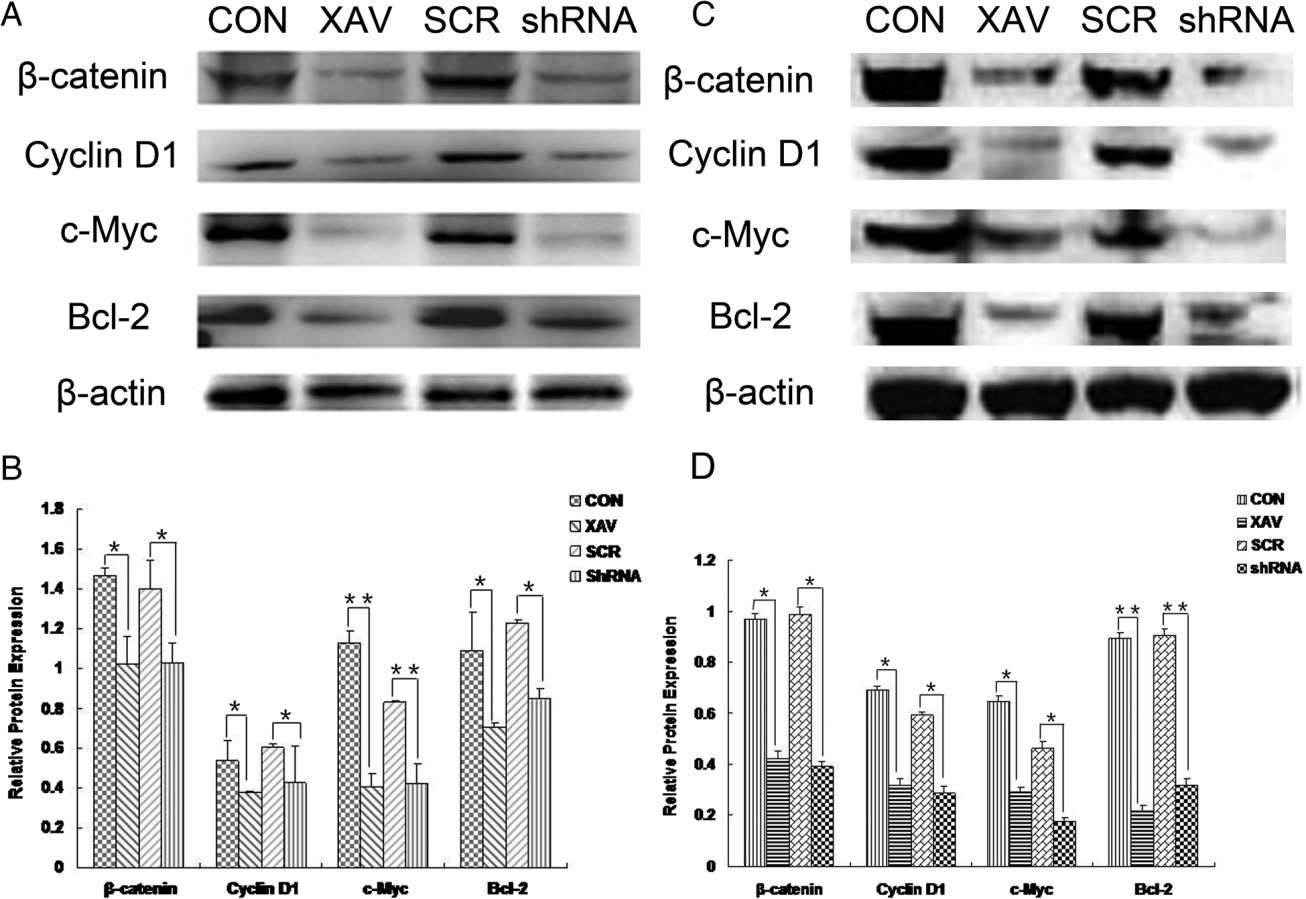
**TNKS1 inhibition altered the expression of anti-apoptotic proteins. A, C.** Western blot analysis of β-catenin, Cyclin D1, c-Myc and Bcl-2 proteins level in SH-SY5Y and SK-N-SH cells untransfected, XAV939 treatment, transfected with TNKS1 shRNA or control shRNA. β-actin was loading control. **B, D.** The bar graph showed mean ± SD of the ratio interest proteins/β-actin band intensity obtained by pooling the results from 3 independent experiments in SH-SY5Y and SK-N-SH cells respectively. CON: control group; XAV: XAV939 group; SCR: SCR group; shRNA: RNAi TNKS1 group. *P < 0.05, **P < 0.01 compared to controls.

It has been reported that XAV939 could inhibit the proliferation of DLD-1 cells growth by attenuating the expression of Wnt signaling [14]. Western blot was performed to determine if TNKS1 inhibition induces apoptosis in SH-SY5Y and SK-N-SH cells and if so whether Wnt/β-catenin signalling and Bcl-2 plays a role. TNKS1 in SH-SY5Y and SK-N-SH cells was inhibited by XAV939 of 1 and 0.5 μM, or by specific shRNA to TNKS1. Following inhibition of TNKS1 in SH-SY5Y and SK-N-SH cells, protein levels of Bcl-2 were both reduced (P < 0.05, Figure 5A, B; P < 0.01, Figure 5C, D). This suggests that TNKS1 inhibition might induce apoptosis in NB cell lines in part by reducing expression of the anti-apoptotic protein Bcl-2. After treating with XAV939 or specific shRNA to TNKS1, we noted that the accumulation of β-catenin reduced as well as Cyclin D1 and c-Myc in both NB cell lines (Figure 5A, C). Quantification analysis revealed these results (P < 0.05, Figure 5B, D). As a result, the anti-apoptotic protein Bcl-2 also decreased.

## Discussion

NB is the most common malignancy of infancy and constitutes 50% of all infantile cancers. NB with higher-grade are quite aggressive and have low cure rates even with combined modality treatments of surgery, radiation and chemotherapy [30]. Understanding the molecular mechanism of NB could help us find key targets which could be exploited efficiently for therapy.

TNKS is a member of the PARP family, which uses NAD + as a substrate to generate ADP-ribose polymers onto target proteins, and results in a post-translational modification referred to as PARsylation [31]. TNKS1 was previously identified as a binding partner for telomerase repeat binding factor 1 (TRF1), which is an important player in the regulation of telomere length at the chromosome ends. It has been shown that TNKS1 expression is up-regulated in several human cancers, and correlates significantly with highly aggressive disease and poor prognosis in some types of cancer, such as breast, colon, and bladder cancer [19-21]. Thus, TNKS1 could be potential therapeutic target for the treatment of malignant NB that overexpressing TNKS1. XAV939, a TNKS1 inhibitor, is synthetized using a chemical genetics approach, and reported to have been used against cancers like colorectal cancers [14,15] and WTK1 human lymphoblastoid cells [32]. However, the antagonism of XAV939 has not been well studied in NB. In the present study we show that inhibition of TNKS1 either by small molecule inhibitor XAV939 or by a specific shRNA decreases the cell viability of NB cell lines (Figure 1). This phenomenon can be explained by induction of apoptosis (Figure 3) or cell cycle arrest (Figure 4).

Furthermore, we assessed the effects of TNKS1 inhibition on cell survival and proliferation in SH-SY5Y and SK-N-SH cells. It has been reported that inhibition of TNKS1 leading to decreased levels of β-catenin by stabilizing Axin and turning off Wnt/β-catenin signaling [14,33]. We showed that SH-SY5Y cells treated with XAV939 induces disaggregation of β-catenin compared to untreated controls (Figure 5), indicating that TNKS1 inhibition leads to degradation of β-catenin. We also found that the downstream target proteins of β-catenin, such as Cyclin D1 and c-Myc, were down-regulated, which demonstrated that Wnt/β-catenin signaling was inhibited. We know that high β-catenin/TCF activity is able to drive cell proliferation during tumor formation by turning on the cell-cycle regulator Cyclin D1 [34], and c-Myc serves as important role in prognosis of NB [35]. The Bcl-2 family of proteins plays a key role in regulation of mitochondrial permeability during apoptosis via intrinsic pathway. In our present study we showed that inhibition of TNKS1 with XAV939 reduced Bcl-2 proteins in SH-SY5Y cancer cells (Figure 5) consistent with the promotion of apoptosis.

Moreover, TNKS1 has been shown to regulate sister telomere separation [36] and mitotic progression [29]. However, there are some glaring discrepancies between the results of Huang et al. and earlier studies on TNKS1 function during mitosis. Huang et al. found that small molecule drug XAV939 didn’t cause mitotic arrest in DLD-1 colon cancer cells, neither RNAi-TNKS1 do. The results were in sharp contrast with other studies [28,29,31]. In the present study we also found that the three NB cell lines, when treated with XAV939, have a prolonged S phase followed by a G2/M cell cycle arrest compared to untreated cells. This discrepancy may be related to different types of cancer and need to be further investigated.

Recently it has been shown that XAV939 inhibits DLD-1 colony formation in an axin-dependent manner [14]. Axin is a concentration-limiting factor in the β-catenin degradation complex and may function more generally as a signal ‘integrator’ in modulating Wnt pathway activity. In our studies, XAV939 as well as shRNA for TNKS1 inhibited SH-SY5Y colony formation in vitro (Figure 2). In conclusion, the present data and previous studies indicate that small molecule inhibitors XAV939 could inhibit the proliferation and colony formation of SH-SY5Y cells by inhibiting TNKS1 might in part through Wnt/β-catenin signaling. But the results are required to be validated in vivo to get a better understanding of the mechanisms involved and the potential role of XAV939 in NB treatment. Moreover, TNKS1 is a protein that participates in both telomere regulation and Wnt/β-catenin signaling, which are essential factors for tumor remedy and recurrence. However, the relationship between the telomere regulation and Wnt/β-catenin signaling need to be further explored. The research will pave the way for NB treatment used by TNKS1 inhibitors.

## Conclusions

In sum, we have shown that inhibition of TNKS1 by XAV939 or RNAi method inhibits the proliferation and induces apoptosis of NB cell lines. One of the related mechanisms may be the inhibiting of Wnt/β-catenin signaling. But more experiments should be carried out to clarify the exact mechanisms. This effect would be expected to promote small molecule targeted therapy in patients with malignant NB.

## Competing interests

The authors declare that they have no competing interests.

## Authors’ contributions

TXH and BSL conceived and designed the experiments. TXH performed the experiments and analyzed the data. HWJ and FY contributed to the data analysis, FJ, TH, CQ and XH has made contribution to the operation of the experiments. TXH and BSL wrote the manuscript, and LY supplied help on the paper writing. All authors have read and approved the final manuscript.
